# The correlation between serum free thyroxine and regression of dyslipidemia in adult males: A 4.5-year prospective study

**DOI:** 10.1097/MD.0000000000008163

**Published:** 2017-09-29

**Authors:** Haoyu Wang, Aihua Liu, Yingying Zhou, Yue Xiao, Yumeng Yan, Tong Zhao, Xun Gong, Tianxiao Pang, Chenling Fan, Jiajun Zhao, Weiping Teng, Zhongyan Shan, Yaxin Lai

**Affiliations:** a Department of Endocrinology and Metabolism, Institute of Endocrinology, Liaoning Provincial Key Laboratory of Endocrine Diseases, The First Affiliated Hospital of China Medical University, China Medical University, Shenyang, Liaoning; b Department of Endocrinology, Shandong Provincial Hospital Affiliated to Shandong University; c Institute of Endocrinology and Metabolism, Shandong Academy of Clinical Medicine, Jinan, Shandong, China.

**Keywords:** dyslipidemia, free thyroxine, high-density lipoprotein, thyroid, triglycerides

## Abstract

Elevated free thyroxine (FT4) levels may play a protective role in development of dyslipidemia. However, few prospective studies have been performed to definite the effects of thyroid hormones on the improvement of dyslipidemia and its components. Thus, this study aims to clarify the association between thyroid hormones within normal range and reversal of dyslipidemia in the absence of intervention.

A prospective analysis including 134 adult males was performed between 2010 and 2014. Anthropometric parameters, thyroid function, and lipid profile were measured at baseline and during follow-up. Logistic regression and receiver operating characteristic (ROC) analysis were conducted to identify the variables in forecasting the reversal of dyslipidemia and its components.

During 4.5-year follow-up, 36.6% (49/134) patients resolved their dyslipidemia status without drug intervention. Compared with the continuous dyslipidemia group, subjects in reversal group had elevated FT4 and high-density lipoprotein cholesterol (HDL-C) levels, as well as decreased total cholesterol (TC), triglycerides (TG), and low-density lipoprotein cholesterol (LDL-C) levels at baseline. Furthermore, baseline FT4 is negatively associated with the change percentages of TG (*r* = −0.286, *P* = .001), while positively associated with HDL-C (*r* = 0.227, *P* = .008). However, no correlation of lipid profile change percentages with FT3 and TSH were observed. Furthermore, the improving effects of baseline FT4 on dyslipidemia, high TG, and low HDL-C status were still observed after multivariable adjustment. In ROC analysis, areas under curve (AUCs) for FT4 in predicting the reversal of dyslipidemia, high TG, and low HDL-C were 0.666, 0.643, and 0.702, respectively (*P* = .001 for dyslipidemia, .018 for high TG, and .001 for low HDL-C).

Higher FT4 value within normal range may ameliorate the dyslipidemia, especially high TG and low HDL-C status, in males without drug intervention. This suggests that a more flexible lipid-lowering therapy may be appropriate for patients with high-normal FT4.

## 1 Introduction

Dyslipidemia is a primary risk factor for cardiovascular disease, which is one of the main causes of death, and results in a worldwide economic burden.^[1]^ Therefore, it is necessary to understand the pathophysiology of dyslipidemia.

Thyroid hormones have several effects on the lipid metabolism. Subjects with overt hypothyroidism or subclinical hypothyroidism have been considered to be accompanied with increased levels of total cholesterol (TC), triglycerides (TG), low-density lipoprotein cholesterol (LDL-C), and decreased high-density lipoprotein cholesterol (HDL-C) levels.^[2,3]^ On the contrary, an unexplained improvement of lipid profile has been observed in patients with hyperthyroidism.^[4]^ Moreover, dyslipidemia in patients with hypothyroidism or subclinical hypothyroidism can be improved after levothyroxine treatment.^[5–8]^ Recently, multiple studies reported that normal high thyroid hormones can also play a protective role in the development of dyslipidemia.^[9–12]^ However, the longitudinal association of circulating thyroid hormones with regression of dyslipidemia remains unknown.

Therefore, we performed this study to investigate the prospective correlation between thyroid hormones within normal ranges and regression of dyslipidemia and its components.

## 2 Methods

### 2.1 Study population

**Figure 1:**
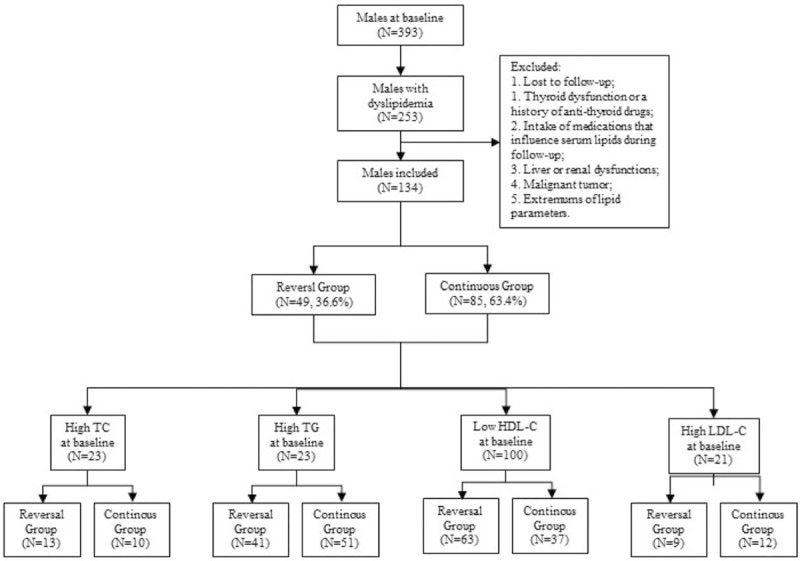
Flow graph of individual recruitment.

We analyzed data from a prospective cohort study of 393 males, aged 40 to 65 years, in urban Shenyang areas of China, in July 2010 and December 2014. Exclusion criteria for these subjects were listed as follows: individuals without dyslipidemia at baseline; a history of thyroid dysfunction or related treatment at baseline or during follow-up; a history of lipid-lowering drugs at baseline or during follow-up; a history of malignancy, liver disease, myocardial infarction, or ischemic stroke; missing communication or unwillingness to participate in the follow-up; and extremums of lipid parameter. In the end, 134 adult males were selected for this study, and follow-up duration was 4.5 years (Fig. 1).

The study was approved by the Ethics Committee of the First Affiliated Hospital of China Medical University and written informed consent was obtained from each participant.

### 2.2 Data collection

All the individuals accepted comprehensive health examinations at baseline and during follow-up by trained researchers. A standardized questionnaire, including demographic and lifestyle characteristics, health status, and medical history was completed for each subject. According to the 2008 Physical Activity Guidelines for Americans, “regular exercise” is defined as moderate-intensity aerobic exercise for at least 150 minutes a week, or vigorous-intensity aerobic exercise for at least 75 minutes a week, or an equivalent combination of moderate- and vigorous-intensity aerobic exercise.^[13]^ At the end of study, we asked each participant if he/she is or has been on a moderate diet during follow-up, such as low-fat diet, vegetarian diet, or other forms. Anyone who asserted to be or have been on a diet is considered as “dietary adjustment.”

As described previously,^[14]^ anthropometric parameters such as weight, height, waist circumference (WC), systolic blood pressure (SBP), and diastolic blood pressure (DBP) were measured, and body mass index (BMI) was calculated.

After fasting for 12 hours, venous blood samples were drawn to determine fasting plasma glucose (FPG), fasting serum insulin (FINS), thyroid function, and lipid profile, and then an oral glucose tolerance test was performed. All the participants were asked to complete fasting blood collection between 7 am and 9 am in order to avoid the effect of blood sampling time on circulating levels of hormones. The homeostasis model assessment for insulin resistance (HOMA-IR) was estimated by the following formula: HOMA-IR = FPG (mmol/L) × FINS (mIU/L)/22.5. Chemiluminescence immunoassay procedures (Roche Diagnostics, Basel, Switzerland) were employed to determine the thyroid-stimulating hormone (TSH), free thyroxine (FT4), free triiodothyronine (FT3), and thyroid peroxidase antibody (TPOAb) in a clinical laboratory. The reference ranges were 0.27 to 4.20 mIU/L for TSH, 12 to 22 pmol/L for FT4, 3.1 to 6.8 for FT3, and 0 to 34 IU/L for TPOAb. Euthyroid status was defined as TSH, FT4, and FT3 within the reference ranges.

The lipid profile including TC, TG, HDL-C, and LDL-C were measured using an auto-analyzer (AU1000; Olympus, Tokyo, Japan). According to the National Cholesterol Education Program Adult Treatment Panel III (NCEP/ATPIII),^[15]^ any of following criteria was defined as dyslipidemia: high TC: TC ≥6.22 mmol/L; high TG: TG ≥1.70 mmol/L; low HDL-C: HDL-C <1.04 mmol/L for males; high LDL-C: LDL-C ≥4.14 mmol/L.

### 2.3 Statistical analysis

In accordance with the lipid status during follow-up, the subjects at baseline were grouped into reversal group and continuous group. The data distribution was assessed by the Kolmogorov–Smirnov test. The variables were displayed as mean ± standard deviations, medians (interquartile range), or counts (percentages) according to their types. Independent *t* test, Mann–Whitney rank sum test, Pearson test, Spearman test, Pearson Chi-squared test, or Fisher exact test was conducted to estimate the relative factors of dyslipidemia and its components depending on the data characteristic. The ability of the FT4 to identify individuals with regression of dyslipidemia was evaluated by the area under receiver operating characteristic (ROC) curves, and the cutoff points were suggested by Youden Index (sensitivity + specificity-1). Statistical analyses were performed by SPSS (version 23.0; IBM Corp, New York, USA). A *P* value less than .05 was considered statistically significant.

## 3 Results

At baseline, 253 of 393 males (64.4%) suffered from dyslipidemia. On the basis of the exclusion criteria, 134 males were included. We compared the baseline characteristics between included and excluded individuals, but no significant difference was observed (Table 1).

**Table 1:**
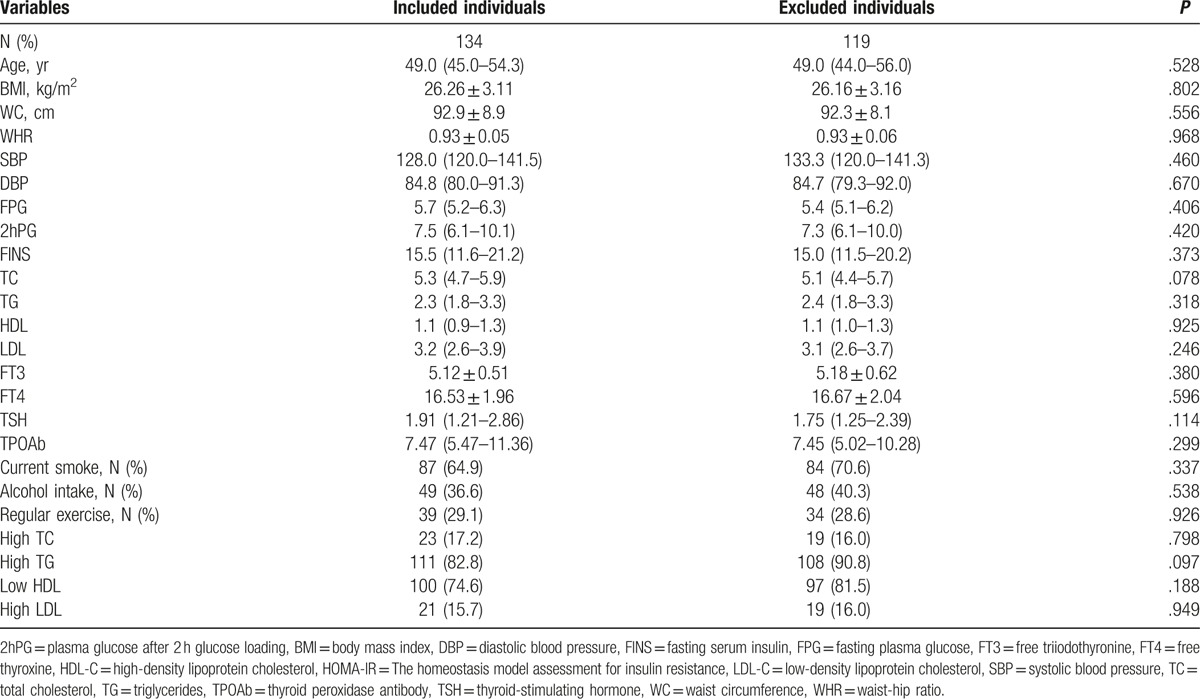
Baseline characteristics of included and excluded individuals.

During an average 4.5-year follow-up, 49 of 134 males with dyslipidemia (36.6%) were returned back to normal lipid levels without drug intervention. Table 2 summarizes the baseline anthropometric and biochemical characteristics associated with the regression of dyslipidemia and its components. Males who regressed from dyslipidemia have lower levels of TC, TG, and LDL-C, and a higher level of HDL-C at baseline. Compared with continuous group, FT4 of reversal group was elevated at baseline. Furthermore, we analyzed the baseline characteristics between reversal and continuous groups of subjects with high TC, high TG, low HDL-C, and high LDL-C. Baseline TG levels of subjects in reversal high TG group were lower than continuous group. And, baseline HDL-C levels of subjects in reversal low HDL-C group were higher than continuous group. Moreover, baseline FT4 levels were significantly elevated in reversal group of patients with high TG or low HDL-C compared with continuous group. Notably, there was no significant difference between reversal and continuous group of dyslipidemia and its components in smoking status, alcohol intake, regular exercise status, and dietary adjustment (Table 2).

**Table 2:**
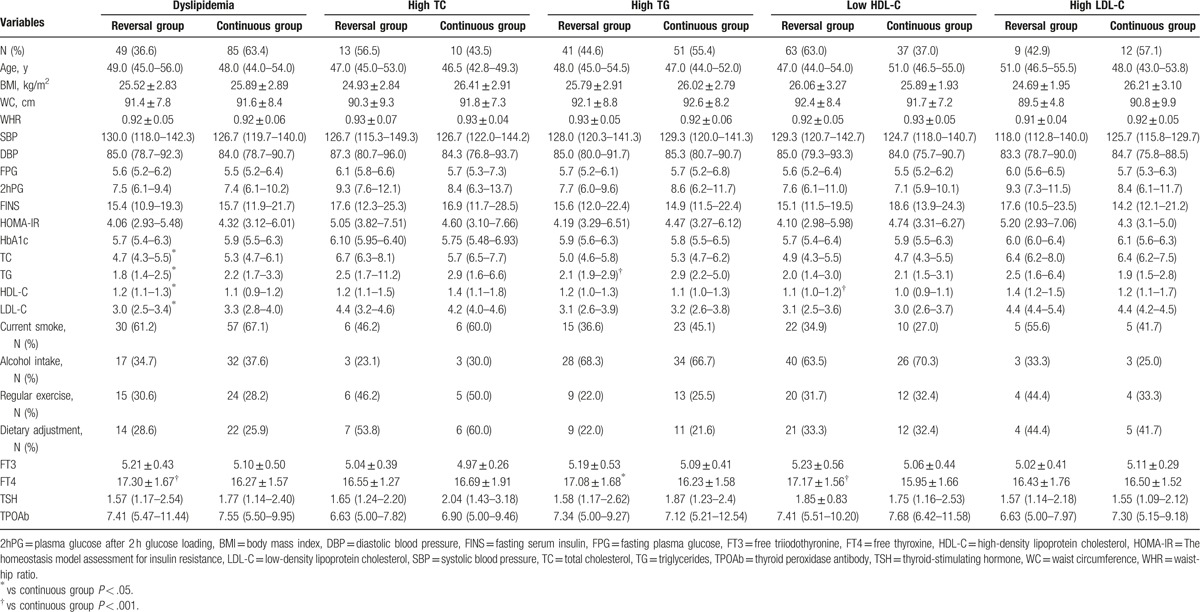
Baseline characteristics of study individuals.

**Figure 2:**
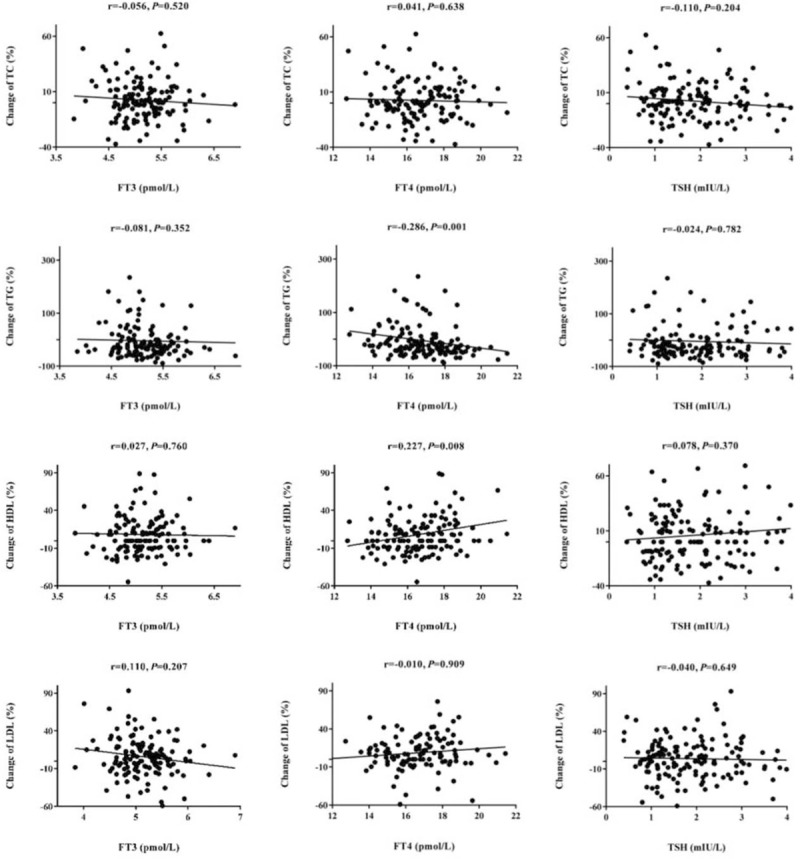
Correlation analysis for thyroid function and percentage change of lipid profile.

Correlation analyses showed that the change percentage of TG is negatively associated with FT4 for high baseline TG patients (*r* = −0.286, *P* = .001), while the change percentage of HDL-C is positively associated with FT4 for low baseline HDL-C patients (*r* = 0.227, *P* = .008). However, no correlation of the other dyslipidemia components with TSH, FT3, or FT4 was observed (Fig. 2).

In order to definite the causal relationship between baseline FT4 levels and regression of dyslipidemia, logistic regression analyses were further performed (Table 3). After adjustment for multivariable, the corresponding relative risks for the reversal of dyslipidemia, high TG, and low HDL-C were 1.897 (1.170–1.490, *P* = .001), 1.422 (1.068–1.894, *P* = .016), and 1.656 (1.233–2.227, *P* < .001), respectively. Results for the other lipoprotein subclasses are summarized in Table 3.

**Table 3:**
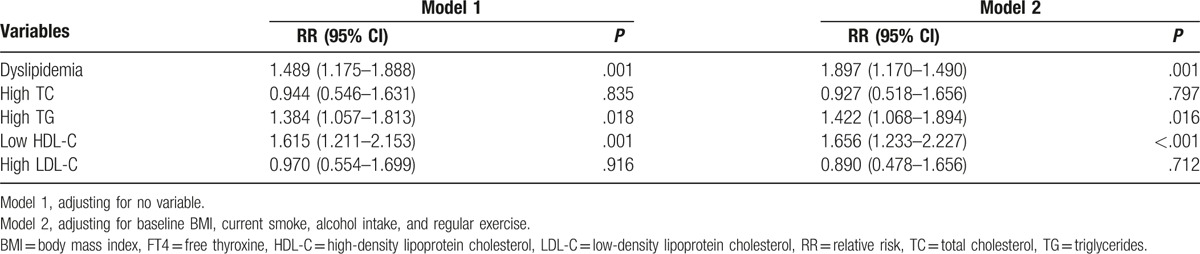
Logistic regression for FT4 and reversal of dyslipidemia and its components.

**Figure 3:**
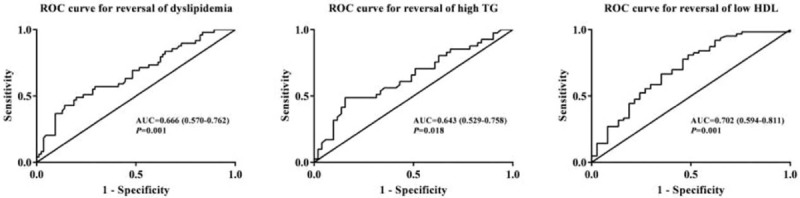
ROC analysis for FT4 in predicting the reversal of dyslipidemia, high TG, and low HDL.

The area under curve (AUC) of the ROC was used to evaluate the ability of baseline circulating FT4 level in predicting the regression of dyslipidemia, high TG, and low HDL-C (Fig. 3). The AUC for predicting reversal of dyslipidemia, high TG, and low HDL-C were 0.666 (0.570–0.762, *P* = .001), 0.643 (0.529–0.758, *P* = .018), and 0.702 (0.594–0.811, *P* = .001). According to Youden index, our research defined the best cut-off values for circulating FT4 levels in predicting regression of dyslipidemia, high TG, and low HDL-C were 17.55, 17.55, and 16.53 pmol/L, respectively.

## 4 Discussion

This study is the first study assessing the impact of thyroid function on the regression of dyslipidemia and its components. To our best knowledge, the disappearance of metabolic syndrome was common (30%), mainly due to a reduction in TG and an increase in HDL-C.^[16]^ In our study, high normal FT4 levels showed an independent association with the regression of dyslipidemia, especially high TG and low HDL-C status, in adult males.

Our findings are in line with several large epidemiological studies. In a cross-sectional study of 2703 euthyroid adults, Rizos et al^[4]^ observed an association between low normal FT4 level and unfavorable lipid profile. Garduno-Garcia et al have reported that FT4 was positively associated with HDL-C, while negatively with HOMA-IR in 3148 individuals. Recently, in another cross-sectional study, a negative association between TG and FT4 within normal range was observed in 2315 middle-age euthyroid males and females.^[17]^ Furthermore, previous studies have reported that high FT4 levels within normal range play a protective role in the development of dyslipidemia,^[10,18,19]^ and levothyroxine replacement can improve the lipid status in subjects with hypothyroidism or thyroidectomy.^[2,7,11,20]^

Therefore, it seems that there are certain underlying pathophysiological mechanisms behind the influence of thyroxine on lipid metabolism. Our study showed a significant positive association between FT4 and HDL-C. Studies in rodents showed that thyroid hormones may promote the synthesis of HDL-C as the primary mechanism for this beneficial effect.^[19,21]^ Furthermore, thyroxine can enhance the reverse cholesterol transport expression, contributing to the improvement of lipid metabolism.^[22]^ In addition, Shin and Osborne ^[12]^ have reported that apart from direct stimulation by thyroid hormones, the LDL-C receptor gene is regulated by SREBP-2, which, in turn, is directly regulated by thyroid hormones.^[23,24]^ It has been reported that alterations of LDL-C in hypo- or hyperthyroidism are correlated to circulating FT4 levels,^[25]^ but we did not observe the association of FT4 with the reduction of LDL-C during follow-up. It may be a result of small sample size for high LDL-C subgroup.

Several methodological limitations of our study should be taken into account. First, our investigation was performed on the basis of a prospective cohort of middle-aged and elderly urban males. Therefore, it may not be generalized for other populations, especially females. Second, we only analyzed thyroid function at baseline. But we excluded individuals with abnormal thyroid function during follow-up, in order to avoid the effects of clinical thyroid diseases on the outcome of dyslipidemia. Third, this study did not collect dietary information of subjects. However, we asked whether subjects had a special dietary adjustment at end, and no significant difference between reversal group and continuous group in dietary adjustment was observed. It does not mean that dietary adjustment has no effect on the reversal of dyslipidemia, for the sample size is small. However, we believed that dietary adjustment has a limited impact on the present results. Moreover, the sample sizes were fairly small in some subgroup analysis. At last, the outcome of blood lipid profile is affected by multiple factors, such as inflammatory state, cytokines, and hepatic metabolism. However, the present study is only an observation of the relationship between dyslipidemia and thyroid function. Therefore, further studies on large scale are needed to uncover the mechanism behind it.

In conclusion, increased serum FT4 is an independent predictor for the regression of dyslipidemia, especially high TG and low HDL-C in adult males, suggesting that a more flexible lipid-lowering therapy may be appropriate for patients with high-normal FT4.

## Acknowledgments

The authors thank the participant and staffs at Institute of Endocrinology of the First Affiliated Hospital of China Medical University, for their involvement in this study. We acknowledge that all authors participated sufficiently in the work and take public responsibility for its content.
